# Human induced fish declines in North America, how do agricultural pesticides compare to other drivers?

**DOI:** 10.1007/s11356-022-22102-z

**Published:** 2022-07-30

**Authors:** Richard Aaron Brain, Ryan Scott Prosser

**Affiliations:** 1 Syngenta Crop Protection LLC, Greensboro, NC USA; 2 School of Environmental Sciences, University of Guelph, Guelph, ON Canada

**Keywords:** Pesticides, Habitat loss, Invasive species, Pollution, Climate change, Anthropogenic factors, Fishes, Freshwater

## Abstract

Numerous anthropogenic factors, historical and contemporary, have contributed to declines in the abundance and diversity of freshwater fishes in North America. When Europeans first set foot on this continent some five hundred years ago, the environment was ineradicably changed. Settlers brought with them diseases, animals, and plants via the Columbian Exchange, from the old world to the new, facilitating a process of biological globalization. Invasive species were thus introduced into the Americas, displacing native inhabitants. Timber was felled for ship building and provisioning for agriculture, resulting in a mass land conversion for the purposes of crop cultivation. As European colonization expanded, landscapes were further modified to mitigate against floods and droughts via the building of dams and levees. Resources have been exploited, and native populations have been overfished to the point of collapse. The resultant population explosion has also resulted in wide-spread pollution of aquatic resources, particularly following the industrial and agricultural revolutions. Collectively, these activities have influenced the climate and the climate, in turn, has exacerbated the effects of these activities. Thus, the anthropogenic fingerprints are undeniable, but relatively speaking, which of these transformative factors has contributed most significantly to the decline of freshwater fishes in North America? This manuscript attempts to address this question by comparing and contrasting the preeminent drivers contributing to freshwater fish declines in this region in order to provide context and perspective. Ultimately, an evaluation of the available data makes clear that habitat loss, obstruction of streams and rivers, invasive species, overexploitation, and eutrophication are the most important drivers contributing to freshwater fish declines in North America. However, pesticides remain a dominant causal narrative in the popular media, despite technological advancements in pesticide development and regulation. Transitioning from organochlorines to organophosphates/carbamates, to pyrethroids and ultimately to the neonicotinoids, toxicity and bioaccumulation potential of pesticides have all steadily decreased over time. Concomitantly, regulatory frameworks designed to assess corresponding pesticide risks in Canada and the USA have become increasingly more stringent and intensive. Yet, comparatively, habitat loss continues unabated as agricultural land is ceded to the frontier of urban development, globalized commerce continues to introduce invasive species into North America, permanent barriers in the form of dams and levees remain intact, fish are still being extracted from native habitats (commercially and otherwise), and the climate continues to change. How then should we make sense of all these contributing factors? Here, we attempt to address this issue.

## Introduction

Anthropological evidence indicates that the Vikings first discovered North America around 1000 Anno Domini (AD), lead out of Greenland/Iceland by the Norse explorer Leif Erikson and arriving in Newfoundland, Canada. However, mass European settlement of the Americas did not occur in earnest until the arrival of the Spanish conquistadors following the landing of Christopher Columbus at San Salvador in the Bahamas on October 12th, 1492. Between 1500 and 1800 AD, European empires including Spain, Portugal, Great Britain, France, and the Netherlands increasingly voyaged across the Atlantic to explore and lay claim to the natural resources and human capital of the Americas during the so-called Age of Exploration at the expense of Indigenous peoples. Canada was originally claimed for France by Jacques Cartier in the 1530 s, Great Britain made several attempts to establish colonies in the modern-day states of Virginia and Maine in the late 1500’s and early 1600’s, ultimately succeeding in Jamestown, and the Dutch laid claim to the Hudson River Valley, New York and lower Canada shortly after. On the brink of failure after losing ~ 80% of its initial population, the Jamestown colony was rescued by the British, who introduced a new variety of tobacco, administered by the merchant John Rolfe (who later married Pocahontas of the Powhatan Confederacy). Consequently, the process of land conversion to agriculture in North America, the removal of native inhabitants, and a surge in biological globalization via the “Columbian Exchange” had begun. First coined by Alfred W. Crosby in 1972, the Columbian Exchange broadly characterizes the transoceanic connectivity established between the old and new worlds facilitating the transport of diseases, animals, and plants (as well as precious metals, goods, and people) (Crosby 1972). This process not only transformed society, culture, politics, and economics in the Americas, but the landscape and environment as well. European colonization and expansion into the new world manifested in the cutting of forests, sowing of fields, rearing of livestock, digging of mines, building of roads and dams, exploitation of natural resources, and the perpetual expansion of “civilization.” As the settler population increased so too did the anthropogenic footprint. There are now over a billion people in the Americas comprising an eclectic assemblage of nations in various states of economic development, as well as environmental degradation/preservation.

The proverbial wave of European settlement swept like a tsunami across the Americas and ineradicably changed the natural landscape in its wake. Following subjugation of the Native American people, dense population centers were established, particularly in areas proximal to water, both freshwater (i.e., lakes and rivers) and marine (oceanic) for the purposes of subsistence and trade, respectively. Among the resources exploited by the new inhabitants, arguably, water has been, and continues to be, the most valuable and vulnerable. Although FEMA (Federal Emergency Management Agency) estimates that 13 million Americans live in areas susceptible to flooding (“flood zones”), Wing et al. (2018) peg that number at nearly 41 million based on higher resolution modeling. Moreover, the National Integrated Drought Information System (NIDIS) indicates that 1/3rd of the USA is currently experiencing severe drought conditions (NOAA 2021). Consequently, development for flood control and water management has become paramount as evidenced by the 91,457 dams (USACE 2021a) and 24,665 miles of levees (USACE 2021b) currently in operation in the USA. Collectively, dams and other obstructions block access to 600,000 miles of rivers (17% of all river miles) across the nation. Moreover, of the ~ 3,200,000 miles (5,200,000 km) of streams in the contiguous USA, 2% are considered to have features of sufficiently high quality to be considered federally protected wild, scenic, and recreational rivers (Benke 1990). Following colonial settlement, total wetland acreage in the conterminous USA has decreased by ~ 53% to around 100 million acres (Dahl 1990). Total water use in the USA was approximately 322 billion gallons per day in 2015, with thermoelectric power, irrigation, and public supply accounting for 40, 37, and 12%, respectively according to the US Geological Survey (USGS 2018). In addition to hydrological intervention, numerous other factors have profoundly influenced water resources in this region, particularly land use change.

Following establishment of the Jamestown colony and formation of the Virginia and Plymouth Companies, chartered by King James I, the mass felling of trees for housing, export to Europe, ship building and provisioning for agriculture had begun. The US Department of Agriculture (USDA 2014) estimates that in 1630, forest land in the USA represented nearly half the total land cover (~ 1,023 million acres; ~ 46%). Subsequently, over a quarter of a million acres have been converted to other uses, primarily agriculture. By 1910, forest land cover had dropped to ~ 34% (~ 754 million acres); however, since then, forest area has been relatively stable, despite a tripling in population (USDA 2014). Since the arrival of the first European inhabitants, agriculture, as a land cover category in the USA, has accounted for a high of 63% of the nearly 2.3 billion total acres in 1949, declining to a current proportion of ~ 50% (USDA 2019). Agricultural lands are now shrinking in the USA and forestry land cover is expanding (USDA 2019) though the resultant implications for biodiversity, water chemistry, soil erosion, and channelization have been significant (Gregory 2001; FAO 2017). While acknowledging the profound influence of historically dominant land use shifts, perhaps the biggest contemporary threat with respect to land use change is the ever-expanding urban footprint. According to the American Farmland Trust, 11 million acres of farmland and ranchland were converted to urban and highly developed land use (4.1 million acres) or low-density residential land use (nearly 7 million acres) between 2001 and 2016 (Freegood et al. 2020). This acreage is equivalent to the annual US farmland devoted to fruit, nut, and vegetable production or ~ 2000 acres a day (Freegood et al. 2020). Thus, habitat loss, as a consequence of land use change, continues to alter streams, rivers, lakes, and coastlines in North America, albeit as a consequence of different socioeconomic drivers.

Analogously, the ships that brought the first European settlers, who transformed the Americas, also brought other species, invasive species, resulting in a sort of parallel Machiavellian Colombian Exchange of ecologically and economically damaging transplants. According to Pimentel et al. (2005), there are approximately 50,000 foreign species in the USA, comprising ~ 2% of all insects and arachnids, ~ 4% of all mollusks (non-marine), ~ 6% of all terrestrial vertebrates, and a staggering ~ 8% of all fish species (Corn et al. 1999). Economically, damages and losses from the introduction of invasive species have been estimated at almost US $120 billion (Pimentel et al. 2005). In the Great Lakes alone, an estimated US $100 million in lost revenue and prevention strategies is attributed to aquatic invasive species annually (Rosaen et al. 2012), and some estimates peg the value as high as US $200 million (The Nature Conservancy 2021). When other side effects are considered such as sport fishing losses, the economic impacts may exceed US $800 million annually (Rothlisberger et al. 2012). This fragile ecosystem, representing the largest source of freshwater on the planet, has experienced the introduction of at least 184 aquatic invasive species from taxa representing viruses, bacteria, protozoa, diatoms, plants, arthropods, mollusks, and fish (Escobar et al. 2018). Consequently, the Non-indigenous Aquatic Nuisance Prevention and Control Act was passed in 1990; however, by the time this legislation was formalized, the enemy was already behind the gates (so to speak).

Subsequent to British colonials landing in Virginia, the Jamestown colony experienced recurring epidemics of typhoid and dysentery between 1607 and 1624 due to water source contamination from feces and urine, killing at least 30% of the population (Earle 1979). These events mark the first documentation of water pollution in the USA. As the colonial population expanded, the demand for natural resources increased. When tobacco was introduced to Virginia in 1610 for export and trade, land had to be cleared and cultivated. Soon, other crops (e.g., cotton and maize [corn]) were sown for subsistence (Gray and Thompson 1933) and agriculture began to expanded across the nation. As the pioneers and homesteaders migrated west in the early 1800s, these “sodbusters” converted approximately 400 million acres (162 million ha) of native prairie grasslands to agriculture (Samson and Knopf 1994). Plowing native vegetation resulted in soil erosion contaminating waterways and other environmental issues epitomized by the dust bowl. Exacerbated by drought conditions and the mechanization of farming, legislation in the form of the Soil Conservation Act was passed to intervene and agricultural science and technology quickly responded. The “green revolution” marked an extraordinary period of food crop productivity growth manifested by advances in crop germplasm, and breeding programs, as well as research and development of pesticides and fertilizers (Noone 1958; Alston et al. 2010; Popp et al. 2013). The first recorded use of a “pesticide” dates back to the eighteenth dynasty in Egypt (1550 to 1292 B.C.E.). The Ebers papyrus describes the use of different substances (fumigants, desiccants, sulfur) to rid a home of pests or to prevent insect pests from consuming grain stores. Later, heavy metals such as arsenic, mercury, and lead were employed to kill pests. However, it was not until the “green revolution” that synthetic chemistry introduced the first pesticides to be applied on a mass scale. Upon their introduction, organochlorine insecticides were hailed as a public health miracle (Roberts et al. 2016), although the ominous, albeit unintended, environmental effects would later be discovered (Carson 1962). The exponential increase in human population has been made possible by the advent of synthetic pesticides and fertilizers; however, there have been unintended consequences, including contamination of water resources with nutrients and agrochemicals (Moss 2010; Pingali 2012). The deleterious effects of organochlorine insecticides in fish, for example, have now been well characterized (Martyniuk et al. 2020). Moreover, long before the advent of the green revolution, the industrial revolution occurred; fueled by coal, and catalyzed by Thomas Newcomen’s steam engine, the age of manufacturing had begun in 1712. Unfortunately, despite the economic transformation, contamination of water resources has been commonplace, exemplified by the burning Cuyahoga River and the enduring toxic legacy of the Love canal. The former incident (which occurred repeatedly) ultimately contributed to the formation of the US Environmental Protection Agency in 1970 and passage of the Federal Water Pollution Control Act of 1972 (commonly referred to as the Clean Water Act). In addition to industrial chemical waste, mining has contributed to heavy metal contamination of water resources (Martin and Platts 1981) and headwater stream obstruction as a result of mountaintop removal (Miller and Zégre 2014).

Commensurate with the global experience, colonization, proliferation, and industrialization of the Americas have had anthropogenic consequences for the climate. Climate change is the consequence of, and the driver for, many of the aforementioned factors. Land use change and pollution clearly have an effect on the climate (Feddema et al. 2005; Pielke 2005; Moss 2010), which clearly has an effect on extreme weather events (Strzepek et al. 2010; Ornes 2018), and implications for freshwater resources, including changes in the distribution of river flows and groundwater recharge (Kundzewicz et al. 2008). Drought frequency is projected to increase in the southwestern states (Strzepek et al. 2010), whereas the frequency and severity of hurricanes are expected to increase in Atlantic and Gulf Coasts (Marsooli et al. 2019). For example, Lake Mead, the largest reservoir in the USA, created by the Hoover Dam on the Colorado River, is currently only 35% full (USBR 2021), having dropped 140 ft since 2000 due to demand and drought. Moreover, Hurricane Harvey was projected to have increased in total precipitation by 20 to 40% as a result of climate change (Risser and Wehner 2017). Declining water quality as a consequence of climate change is likely to increase water withdrawals, pollution from diffuse sources (via higher runoff and infiltration from heavy precipitation), malfunctioning of water infrastructure during floods; and overloading the capacity of water and wastewater treatment plants during extreme rainfall (Kundzewicz et al. 2008).

While the first British colony in Jamestown sparked the agricultural transformation of North America, it was the subsequent colonies in Plymouth and Massachusetts Bay that launched commercial fishing in the early seventeenth century. Although the most indelible examples of human exploitation in the USA include the bison (*Bison bison*), prairie dog (*Cynomys spp.*), and passenger pigeon (*Ectopistes migratorius*), lesser known are the many examples of fish, where 57 taxa have become extinct between 1898 and 2006 (Burkhead 2012). As described by Regier et al. (1999), fish communities near larger settled areas in the Laurentian Great Lakes “hit the wall” ecologically beginning in the late 1800s as a result of over-fishing. Although overfishing remains near all-time lows in the USA at present (NOAA 2021), the historical impact has been significant (Smith 1968; Regier et al. 1999; Jackson et al. 2001). The first population of fish to disappear from North America was the Atlantic salmon (*Salmo salar*) of Lake Ontario (Burkhead 2012). Lake sturgeon (*Acipenser fulvescens*), lake herring (*Coregonus artedi*), lake whitefish (*Coregonus clupeaformis*), and other species were also severely impacted by commercial fishing, prior to the collapse of the industry in the 1960s following the introduction of the sea lamprey (*Petromyzon marinus*) into Lake Ontario (Smith 1968). Progressive shifts in species succession have therefore occurred as a result of exploitation (Smith 1968). Currently, there are over 1200 species of native freshwater fishes in North America, 75% of which occur within the USA, including 53 families, 214 genera, representing 8.9% of the Earth’s freshwater fish diversity (Warren and Burr 1994; Nelson et al. 2004). Approximately 1 in 7 Americans currently engage in recreational fishing, and nearly 30 million are paid license holders (Statista 2021).

The impact European colonization has had, and continues to have, on water resources of the Americas is extensive; there is nary a stream, river, lake, or pond that has not been influenced by anthropogenic activity. In the conterminous USA, 86% of streams have experienced altered flow due to human impacts on watershed hydrology (Carlisle et al. 2011), and 46% of the nation’s river and stream length is in poor condition (USEPA 2016a, b). These anthropogenic factors and many others also impact the Great Lakes, which contain a fifth of the world’s freshwater (Jenny et al. 2020). Therefore, it is not surprising that many species endemic to these habitats have experienced considerable human-induced losses, particularly fishes. As indicated by Walsh et al. (2011), approximately 40% of freshwater fish in the USA are imperiled or presumed extinct; 140 species are listed as threatened or endangered (USFWS 2021a). Moreover, the rate of extinction of freshwater fishes in North America is estimated to be 877 times the historical background rate (Burkhead 2012). Specific threats to this fauna include “habitat destruction, introduced species, altered hydrology, pollution, sedimentation, disease, parasitism, over-exploitation, and other factors” (Walsh et al. 2011). But which of these is the most significant driver of fish declines relatively speaking? A fairly recent meta-analysis conducted by Stehle and Schulz (2015) suggested that agricultural insecticides were threatening surface waters at the global scale. However, how accurate is the conclusion when framed within the broader context of other contributing anthropogenic factors, particularly when viewed through a historical lens? The objective of this manuscript is to attempt to address this question by systematically evaluating the relative influence of the most prominent drivers for species declines in North American freshwaters, focusing specifically on fishes. The analysis follows the same conceptual framework as previous employed for birds (Brain and Anderson 2019) and other wildlife (Brain and Anderson 2020). The hypothesis being tested is that among myriad of anthropogenic factors contributing to fish declines, contextually, pesticides are not a significant driver; the null hypothesis being that they are.

## Potential drivers of freshwater fish declines

### Habitat loss

The most common cause of the extinction of freshwater fish species over the last 100 years in North America has been habitat loss, which also represents the greatest threat to existing freshwater fish species (Dextrase and Mandrak 2006; Deinet et al. 2020; NOAA Fisheries 2021). A large body of literature outlines activities that cause habitat degradation and mechanisms by which these activities cause changes in fish habitat (Gregory and Bisson 1997; Gibson et al. 2005; Dudgeon et al. 2006; Smokorowski and Pratt 2007; Finigan et al. 2018). The other drivers of freshwater fish decline to be discussed herein (i.e., dams/obstructions, invasive species, over-exploitation, climate change, pollution) are ultimately all factors that potentially interact to degrade habitat for freshwater fishes (Gregory and Bisson 1997; Dudgeon et al. 2006). Certain activities directly cause habitat destruction (e.g., draining wetlands, dam construction, mining sand or gravel) by immediately changing the physical structure of habitat, while other activities indirectly result in fish habitat destruction (e.g., harvesting forests in headwaters of watershed, removal of riparian vegetation, conversion of land for agricultural production or human settlements in the upstream catchment) by causing changes in inputs (e.g., sediment, nutrients, organic matter) to the aquatic system (Naiman et al. 1995; Dudgeon et al. 2006).

Walsh et al. (2011) rank habitat destruction and modification, including dam construction, channelization, mining, clearing of natural forests for agriculture, urban development, and other intensive land-use practices, among the most important threats to fishes in freshwater habitats. Gregory and Bisson (1997) outlined six components of stream ecosystems that are critical for the conservation of fish habitat: channel structure, riparian structure, hydrology, sediment input, water quality, and exogenous inputs (e.g., toxicants, exotic species). The primary purpose of changing the channel of streams and rivers has been to control flooding, but channelization can also be used to improve navigability of the waterway and to drain wetlands (Schoof 1980; King et al. 2010). Over 300,000 km of streams and rivers in the USA have undergone channelization (Schoof 1980). Changes to the structure of the channel can result in changes to the floodplain, pool and riffles, presence of large woody debris, stream substrate, and the hyporheic zone (Duvel et al. 1976; Smokorowski and Pratt 2007; Dutta et al. 2018; Sanders et al. 2020). These alterations caused by channelization impact fish populations through loss of overwintering habitat, loss of refugia from high flows, loss of rearing sites for larval and juvenile fish, loss of cover from predators, and reduced storage of sediment and organic matter (Brown and Hartman 1988; Swanson et al. 1990; Booth 1991; Gregory et al. 1991; Ralph et al. 1994; Gregory and Bisson 1997). Land use change in the catchment can affect fish habitat in streams and rivers, but removal of riparian vegetation can have a considerably greater impact on fish habitat (Wesche et al. 1987; Pusey and Arthington 2003; Knight and Bottorff 2020). The loss of riparian vegetation impacts fish habitat by causing a reduction of cover from predators and high flow events, reduction in food items drifting on the surface, reduced allochthonous inputs, reduced channel stability, increased streambank erosion, increased water temperatures, altered nutrient inputs, and altered primary and secondary production (Gregory et al. 1991; Bilby and Bisson 1992; Naiman et al. 1992; Simon and Collison 2002; Sweeney et al. 2004; Dosskey et al. 2010). A national inventory on the status of riparian land in Canada and the USA has not been created, but Hirsch and Segelquist (1978) estimated that 70 to 90% of riparian area in the USA had been altered. Swift (1984) reported that woody riparian plant communities covered 30 to 40 million hectares of land in the contiguous USA; however, approximately 70% of that area has been converted to non-forested land. The percentage of forested riparian land that has been converted is as high as 95% in some regions of the USA (Swift 1984).

Along with changes to channel structure and riparian area, hydrology is an important component of fish habitat. Dams, channelization, removal of riparian vegetation, and land use change in the upstream catchment alter the hydrology of streams by changing stream discharge, the speed of fluctuations in flow, and the magnitude of peak and low flows (Schilling et al. 2010; Lei and Zhu 2018; Ni et al. 2021). These hydrological changes can impact fish by reducing availability of food, reducing primary and secondary production, accelerating erosion of streambanks, increasing vulnerability to predation, involuntary downstream movement, and increased competition for forage (Bauersfeld 1978; Jensen and Johnsen 1999; Bunn and Arthington 2002; Schmutz et al. 2014; Yang et al. 2020). Altered hydrology, catchment land use, and riparian structure can also increase the transport of sediment into streams and river due to increased terrestrial surface erosion and collapse of bank material (Sutherland et al. 2002; Liébault et al. 2005; Dos Reis Oliveira et al. 2018). Elevated sediment inputs can damage spawning areas, reduce survival of larval and juvenile fish, inundate pool habitat, reduce primary and secondary production, and disrupt foraging for some fish species (Nerbonne and Vondracek 2001; Sutherland et al. 2002; Henley et al. 2010; Kemp et al. 2011). An important contributor to both changes in hydrology and sediment input is conversion of land in the catchment (Matheussen et al. 2000; Allan 2004). Since colonial settlement, the land use across North America has changed considerably. For example, human disturbance on the landscape of the eastern USA was minimal in 1650, but 30% of the landscape had been disturbed by 1850, and human disturbance increased to 93% by 1920 and 100% by 1992 (Steyaert and Knox 2008). Sleeter et al. (2013) investigated the more recent change in land-cover in the conterminous USA from 1973 to 2000. They found that the largest decline among the different land-cover classes was in forest cover (− 97,273 km^2^; − 4.2%) and the greatest increase was observed in developed land cover (77,529 km^2^; 33%) (Sleeter et al. 2013). This level of change in land use has considerable consequences for lotic systems within the catchment and the habitat these systems provide for fish populations.

The loss of fish habitat due to changes in land use is not isolated to streams and rivers, and habitat has also been lost in lentic ecosystems (e.g., lakes, wetlands). Wetland land cover in the conterminous USA has proportionally declined to the same extent as forest cover from 1973 to 2000 (− 13,639 km^2^; − 4.5%) (Sleeter et al. 2013). Davidson (2014) estimated that since 1900, 36.5% of wetlands have been lost in North America. Wetland ecosystems are used by many fish species to spawn, forage, shelter, and as a nursery area. For example, Jude and Pappas (1992) found that 82 species of fish in the Great Lakes, out of 113 species included in their study, either utilized and/or were closely associated with coastal wetlands. This illustrates the importance of wetlands as fish habitat and the consequences to fish populations and diversity from loss of wetlands. The quality of fish habitat in lake ecosystems has also been impacted by changes in land use. For example, Finigan et al. (2018) observed significant changes in the fish community in 22 in-land lakes in the province of Ontario that corresponded with significant changes in land use around the lakes and significant increases in water temperature. Giacomazzo et al. (2020) found that nutrient and sediment loading due to land use changes in the catchment caused a decline in submerged aquatic vegetation, which led to a decline in a productive yellow perch fishery in Lake Saint-Pierre, the largest fluvial lake of the St. Lawrence River. Jenny et al. (2020) warned of the decline of the world’s large lakes and identified that littoral shoreline modification was one of the six major threats to the world’s large lakes. The other threats being nutrient loading, climate change, acidification, invasive species, and excessive harvesting of fish (Jenny et al. 2020). There are a number of additional studies reporting similar negative impacts as a result of changes in land use within lake catchments on fish habitat and fish populations (Sly 1991; Evans et al. 1996; White and Rawles 2006; Eyles et al. 2013).

The leading cause of freshwater fish decline in North America is habitat degradation and loss. As initially discussed above, there are several human activities that are causing this loss, with changes in catchment land use being a leading cause. In the next section, the specific activity of the construction of dams and other obstructions across streams and rivers and their contribution to the degradation of fish habitat will be discussed.

### Dams/obstructions

**Fig. 1:**
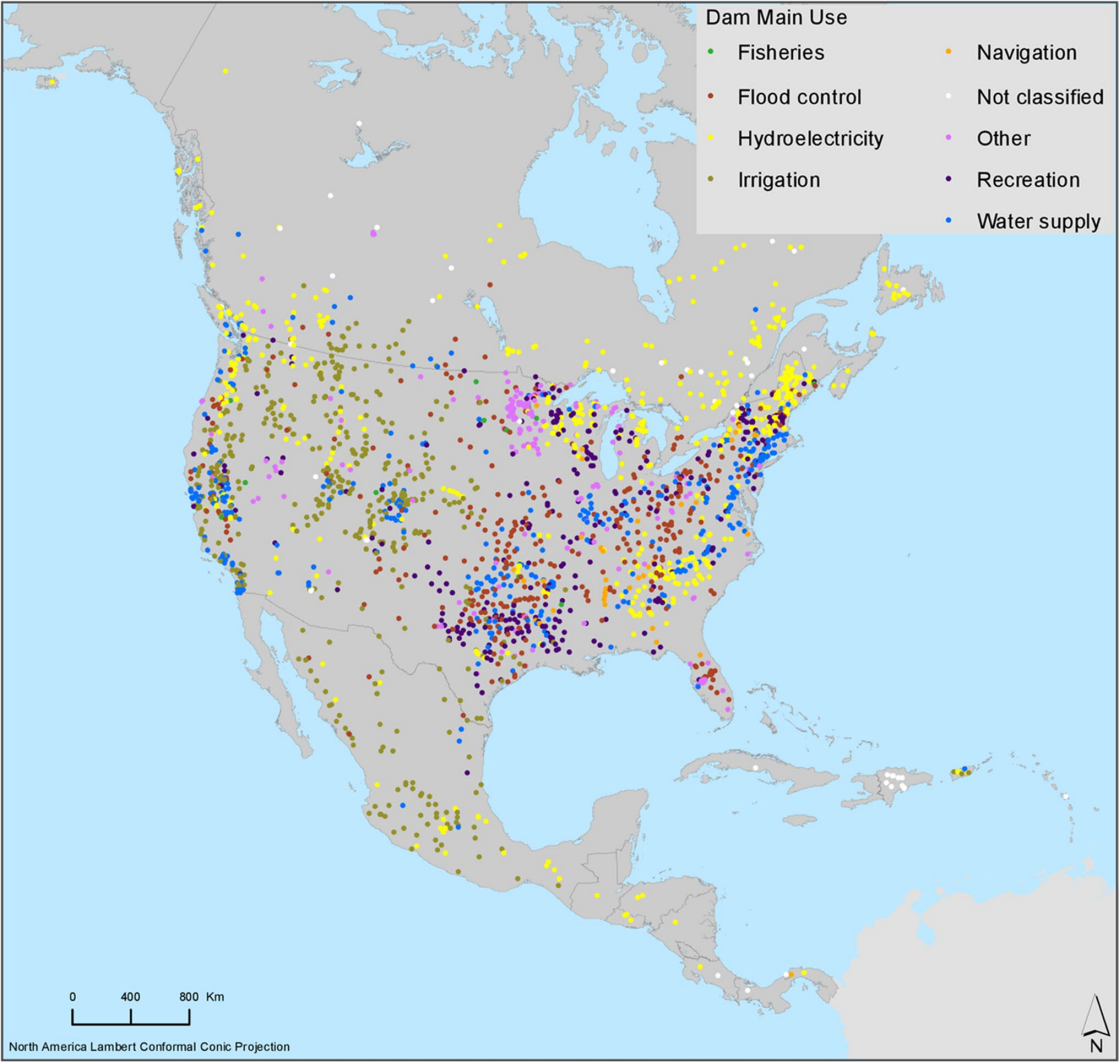
Dams with reservoir storage capacity > 0.1 km.^3^ in North America (Lehner et al. 2011)

Dams and barriers have been constructed across rivers to provide sources of renewable energy, ensure water security, facilitate transportation along waterways, and/or prevent flooding. In the USA, there are more than > 75,000 large dams and > 2.5 million barriers across rivers (USACE 2018; NOAA Fisheries 2021) (Fig. 1). Consequently, the movement of fish has been restricted in > 950,000 km of rivers and streams in the USA (NOAA Fisheries 2021). Limiting the movement of fish restricts the dispersal of species and impedes migration required for feeding and spawning (Herbert and Gelwick 2003; Fuller et al. 2015; Carvajal-Quintero et al. 2019; Barbarossa et al. 2020). The number of stream segments in the conterminous USA has increased 801% due to the construction of dams and 79% of stream length is disconnected from the ocean or Great Lakes (Cooper et al. 2017). Restriction of movement and fragmentation of habitat caused by dams and barriers have been an important driver in the decline of a variety of freshwater fish species in North America. In California, 83% (100/120 species) of inland fish species are either listed as threatened or endangered under state or federal legislation or designated as a species of special concern by the state due to their population being in decline (Moyle et al. 2011). Moyle et al. (2011) determined that dams were the greatest factor of extinction risk for a quarter of the at-risk species identified in California. Habitat fragmentation and water depletion in the Great Plains region of the USA have resulted in a decline or extinction of 84% of the 49 endemic Great Plains fish species (Perkin et al. 2015). A major driver of habitat fragmentation and water depletion in the Great Plains is streams containing > 19,000 barriers, with larger dams causing up to an 88% reduction in stream flow, making the flow of these streams among the most regulated in the world (Lehner et al. 2011; Costigan and Daniels 2012; Perkin et al. 2015). Salmonid species have been particularly impacted by the damming of rivers. This anadromous group of fish requires that adult fish be able to migrate upriver to spawn and requires juvenile fish be able to migrate back downriver, dams, and other barriers hinder both of these stages of the salmon life cycle (Banks 1969; Raymond 1979; Booth 1989; Ferguson et al. 2011). For example, dams and barriers have played a central role in the extirpation of approximately 30% of the salmon populations on the Columbia River, which originates in British Columbia and flows between Washington and Oregon on the way to the Pacific Ocean, while remaining populations have seen significant declines since the 1970s (Gustafson et al. 2007; Ferguson et al. 2011). There is a relatively large body of literature that has been published over the last 50 years that report dams and barriers having a significant impact on fish species that occupy lotic systems (Marts and Sewell 1960; Miller et al. 1989; Haro et al. 2000; Pringle et al. 2003; Limburg and Waldman 2009). This impact has also not been isolated to anadromous species because dams and barriers not only inhibit migration along rivers, but as mentioned in the previous section, they also degrade fish habitat (Cumming 2004; Hayes et al. 2006; Cooper et al. 2017; Wright and Minear 2019). Some of the ways that dams degrade fish habitat are through inundating habitat above dams, changing temperature regimes below dams, reducing seasonality of flow, changing channel dimensions, and changing the distribution of riffles and pools (Bunn and Arthington 2002; Cooper et al. 2017; Barbarossa et al. 2020). However, as discussed above, dams are only one of several human activities that can lead to the degradation of habitat for freshwater fish, which then leads to the decline of these species (Walsh et al. 2011).

### Invasive species

**Fig. 2:**
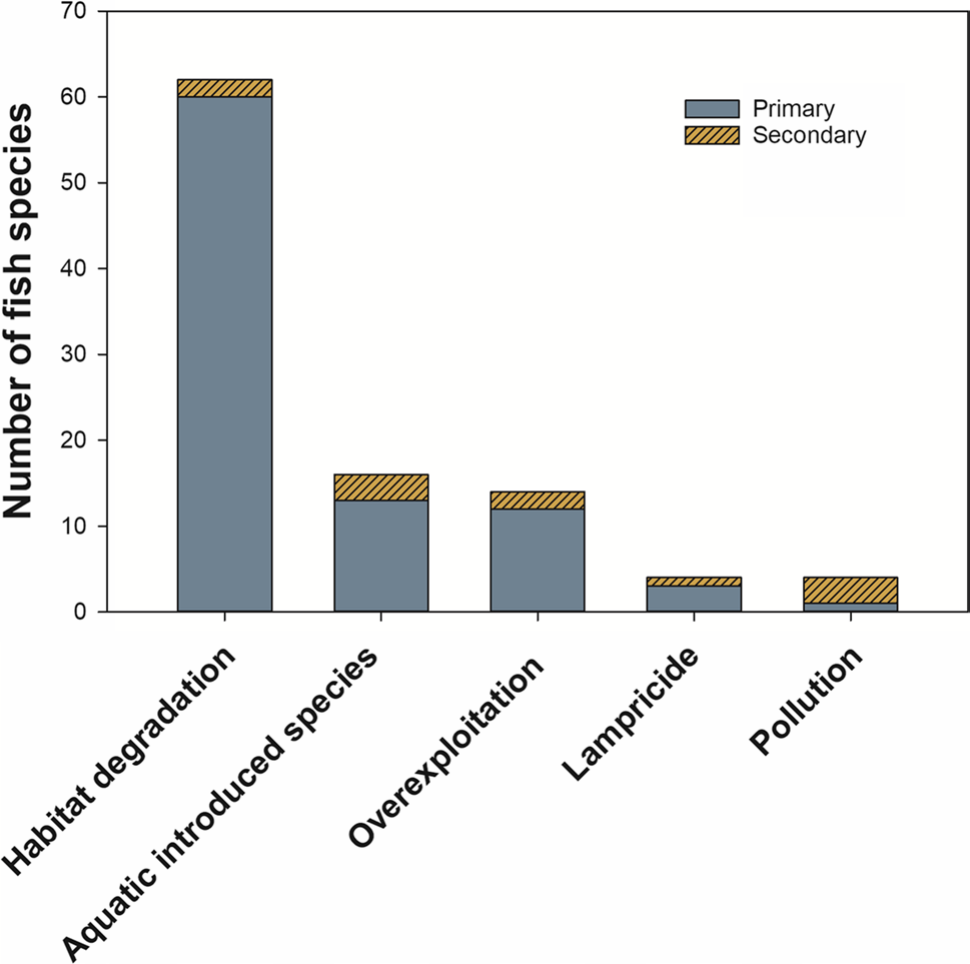
The primary and secondary threats to freshwater fish species that have been identified as at risk of extinction in the Laurentian Great Lakes according to Mandrak and Cudmore (2010)

The estimated median annual cost in ecosystem services of introduced aquatic species in the US waters of the Great Lakes is US $138 million (Rothlisberger et al. 2012). Haubrock et al. (2021) estimated that invasive freshwater fish specifically have cost an economic loss of ≥ US $25 billion, not to mention contributing to the extinction of priceless indigenous fish species. After habitat loss, invasive species are the next largest contributor to the decline in freshwater fish populations in North America (Gregory and Bisson 1997; Dextrase and Mandrak 2006; Moyle et al. 2011; Walsh et al. 2011; Arthington et al. 2016; Escobar et al. 2018; Deinet et al. 2020; Jenny et al. 2020; NOAA Fisheries 2021) (Fig. 2). Introduced species impact native freshwater fish species directly through predation or competition, or indirectly, through spreading disease or the disruption of food webs (Mandrak and Cudmore 2010; van der Veer and Nentwig 2015; Gallardo et al. 2016). The US Fish and Wildlife Service estimates that a third of all species protected under the Endangered Species Act are considered to be at risk, at least in part, due to displacement by, competition with, and predation by invasive species (USFWS 2012). Invasive species are also the second leading cause of the extinction of fish in North America (27 out of 40 species) over the last century, after habitat destruction (Miller et al. 1989).

The Committee on the Status of Species at Risk in Canada (COSEWIC) reported that alien invasive species were a primary factor in the extinction of four of the freshwater fish species that have gone extinct in Canada, with invasive species being the secondary factor to the other extinct species (Dextrase and Mandrak 2006). The largest number of freshwater fish species listed as threatened or endangered in Canada are located in the Great Lakes-Western St. Lawrence ecological area. Alien invasive species are responsible for the listing of greater number of these fish species in this ecological area than any other region in Canada (Dextrase and Mandrak 2006). In the Great Lakes basin, 35 non-indigenous species have successfully established reproducing populations, while 34 species have been observed in the basin, but it is not clear whether they have established reproducing populations (Mandrak and Cudmore 2010). An example of an introduced freshwater fish species that has impacted native species is the rainbow smelt (*Osmerus mordax*). Rainbow smelt was introduced to the Great Lakes in 1912 as a forage fish; by 1930, they had invaded all five Great Lakes, and they are now present in inland lakes as far north as the Lake Winnipeg-Nelson River drainage. This single introduced species is considered a threat to four species listed as threatened and were an important factor in the extinction of blue pike (*Sander vitreus glaucus*) in Canada (Franzin et al. 1994; Wright 2002). Elsewhere, there is evidence that throughout the Mississippi River Basin, relative abundance of the invasive silver carp (*Hypophthalmichthys molitrix*) has increased while the relative abundance and condition of native planktivores (bigmouth buffalo *Ictiobus cyprinellus* and gizzard shad *Dorosoma cepedianum*) has declined (Phelps et al. 2017) and modeling suggests that bighead (*Hypophthalmichthys nobilis*) and silver carp could cause a similar disruption of food webs in the Great Lakes (Zhang et al. 2015).

Non-indigenous fish species are not the only invasive group of organisms that can have been shown to have a negative effect on native fish communities (Strayer et al. 1999; Higgins and Zanden 2010; Yan et al. 2011). An important example is the introduction of dreissenid mussels (e.g., zebra mussels *Dreissena polymorpha*, quagga mussels *Dreissena bugensis*), described as ecosystem engineers, to freshwater ecosystems in North America. The establishment of dreissenids can result in changes to structure and function of freshwater ecosystems (Strayer et al. 1999; Zhu et al. 2006; Hansen et al. 2020). These invasive mussels remove large quantities of suspended sediment, bacteria, phytoplankton, and micro-zooplankton from the overlying water, leading to increased water clarity and light transmittance, which shifts production and biomass from the pelagic to benthic food webs (MacIssac 1996; Strayer et al. 1999; McNickle et al. 2006). For example, the establishment of zebra mussels in the Great Lakes has resulted in the significant decline of *Diporeia* zooplankton, which are an important component of the diet of a number of fish species, e.g., alewife (*Alosa pseudohargengus*; also an introduced fish species) and lake whitefish (*Coregonus clupeaformis*) (McNickle et al. 2006; Pothoven and Madenjian 2008). This change in the food web has affected several different fish species in the Great Lakes. For example, the growth of juvenile lake whitefish has reported to have declined in a number of the Great Lakes since the establishment of zebra mussels (Fera et al. 2015). The effect of dreissenid species on freshwater lake food webs is an example of how invasive species can indirectly adversely affect native fish communities.

The sea lamprey (*Petromyzon marinus*) is an example of an introduced species that has had a direct impact on native fish species, as the lamprey parasitizes larger body fish, e.g., lake trout (*Salvelinus namaycush*), lake sturgeon (*Acipenser fulvescens*), lake whitefish, and walleye (*Sander vitreus*) (USFWS 2021c). The sea lamprey was introduced to the western Great Lakes through the construction of the Welland Canal, which connected Lake Erie with Lake Ontario. The decline of lake trout is a clear example of the magnitude of effect that sea lamprey have had on native fish species. The arrival of sea lamprey aligned with the drastic decline and near extirpation of lake trout populations in Lakes Huron, Michigan, and Superior starting in the 1940s and 1950s (Coble et al. 1990; Hudson and Ziegler 2014). The introduction of sea lamprey had a cascading effect on the fish communities in the western Great Lakes. For example, as lake trout populations declined due to the parasitism of lamprey, the lamprey switched to other native species, e.g., white sucker (*Catostomus commersoni*) (Henderson 1986). Sea lamprey predation was not the only factor that contributed to the precipitous decline of lake trout in the western Great Lakes; overfishing before the introduction of lamprey also played a role (Walters and Steera 1980; Eshenroder 1992). Sea lamprey had a greater impact on Lake Michigan than any other introduced species (Wells and McLain 1973). It was estimated that sea lamprey eliminated nearly 2 million kg of fish per year from Lake Michigan during the 1950s (Smith 1968). The greatest impact was to deepwater cisco (*Coregonus johanna*), as lake trout had been nearly extirpated from Lake Michigan at that time (Wells and McLain 1973). The destruction of the predatory fish by the sea lamprey also contributed to the invasion of the introduced alewife, which also had an impact on the fish communities in the Great Lakes (Smith 1970). For example, the deepwater cisco was extirpated from Lake Michigan and Lake Huron due to the introduction of alewife, introduction of sea lamprey, and overfishing (Wells and McLain 1973). In the following section, the contribution of overexploitation to the decline of fish species in North America will be discussed in more detail.

### Overexploitation

Overexploitation of specific fish species began upon European colonization of North America. Certain species were harvested in large quantities due to their nutritional value to colonizers, e.g., lake herring (*Coregonus artedi*), lake whitefish, sturgeon species (*Acipenser* spp., *Scaphirhychus* spp.), and lake trout (Hudson and Ziegler 2014). For example, selective overfishing of preferred species has contributed to considerable changes in the fish community and the extirpation of a number of species from the Laurentian Great Lakes over the last 200 years (Smith 1968; Regier et al. 1999). The first two fish populations to collapse in the Great Lakes due to overfishing was lake herring and lake whitefish (Smith 1968). Lake herring stocks collapsed in Lake Erie in the 1920s, but before the collapse, the annual harvest could range from 9 to 22 million kg in Lake Erie (Van Oosten 1930; Smith 1968). Following the collapse, lake herring continued decline in Lake Erie, reaching near extirpation from the lake in the 1960s (Smith 1968). Lake whitefish populations collapsed in Lake Huron as a result of the introduction of the deep trap net in the late 1920s (Baldwin and Saalfeld 1962).

It is important to note that overfishing of a specific fish species cannot only impact the population of that desired species but also other species through the destruction of fish habitat or disruption of food webs. For example, lake sturgeon (*Acipenser fulvescens*) were abundant in all of the Great Lakes before 1900, but this large fish would often disrupt fishing for more valuable species in the nearshore area by damaging fishing gear. For this reason, the lake sturgeon were deliberately killed, not as a food source, but to simply prevent them for disrupting the harvest of valuable fish species (Harkness and Dvmond 1961; Baldwin and Saalfeld 1962). Over 450,000 kg of lake sturgeon was removed from each of the upper Great Lakes in the years leading up to the twentieth century (Baldwin and Saalfeld 1962). The populations of this species were greatly diminished in the Great Lakes by the 1920s, to the point that fishing was prohibited (Smith 1968). Lake sturgeon populations have not recovered, and this species remains scarce in the Great Lakes to this day.

In other North American watersheds in the middle and end of the nineteenth century, different species of sturgeon began to decline as a result of commercial harvest for caviar (from eggs), meat, isinglass gelatin (from swim bladders), and oil (Saffron 2004). The Atlantic sturgeon (*Acipenser oxyrhynchus*) populations in the rivers along the east coast of the USA were decimated by the start of the twentieth century after 30 years of harvesting (Secor and Waldman 1999; Waldman and Secor 1999). White sturgeon (*Acipenser transmontanus*) populations in rivers along the west coast met a similar fate by the start of the nineteenth century but in a shorter period of time (Skinner 1962; Waldman and Secor 1999). Atlantic and white sturgeon are currently listed as endangered under the Endangered Species Act (ESA) in the USA and have been extirpated from a number of rivers that they originally inhabited (USFWS 2021b). Despite the historic precedent, one of the two species of sturgeon not currently listed under the ESA in the USA is being impacted by overexploitation. Overfishing is adversely affecting the growth and recruitment of shovelnose sturgeon (*Scaphirhynchus platorynchus*) in the upper Mississippi River (Colombo et al. 2007; Thornton et al. 2019). Overexploitation of freshwater fish populations has declined due to conservation and management efforts, but it has not ceased.

### Climate change

Climate change is a human-induced threat to all ecosystems on Earth. Freshwater ecosystems will be affected by changes in the Earth’s climate (Millenium Ecosystem Assessment 2005; Hering et al. 2010; Woodward et al. 2010; Collingsworth et al. 2017; Hasler et al. 2018). Consequently, many freshwater fish populations in North America are at risk due to climate change (Comte et al. 2013; Comte and Olden 2017). Models developed by Chu et al. (2005) predict that cold water fish species will be extirpated from their present range in Canada while warm water species may expand their range. To illustrate the potential magnitude of change that may occur to freshwater fish communities due to climate change, under a number of different climate change scenarios, lakes located in the Arctic may represent suitable habitat for smallmouth bass (*Micropterus dolomieu*), a warm-water fish species, by 2100 (Sharma et al. 2007). This scenario has frightening consequences for cold-water fish species in North America. Schinder (1997) outlines in detail the many ways in which a warming climate will impact freshwater ecosystems in North America. Climate change will effect hydrology, physical features, and chemical characteristics of lakes and streams in North America (Schinder 1997). A warming climate may also exacerbate other factors that adversely affect freshwater fish populations. The effects of eutrophication and harmful algal blooms, which will be discussed in more detail in the “Pollution” section, may be worsened due to reduced flow and reduced oxygen saturation of water caused by a warming climate (Schinder 1997; Moss et al. 2011; O’Neil et al. 2012; Salmaso et al. 2017). Several studies have already observed that warming temperatures will exacerbate oxygen depletion due to eutrophication (Foley et al. 2012; Li et al. 2018; Budnik et al. 2021). For example, Foley et al. (2012) found that climate change and eutrophication increased hypolimnetic anoxia significantly over time from 1968 to 2008 in a small temperature lake, which will have negative consequences for fish populations in these types of lakes. While habitat degradation due to human activity was the greatest threat to freshwater fish species in the previous century, climate change will likely be the greatest threat in the next century, particularly given the pace that the climate is warming.

### Pollution

After channel structure, riparian structure, hydrology, and sediment input, the last two components critical to the conservation of fish habitat outlined by Gregory and Bisson (1997) were water quality and exogenous inputs. Gregory and Bisson (1997) considered the introduction of invasive species as an exogenous input, which was discussed above, but this section will focus on inputs from prominent human activities that can alter water quality, e.g., industry, wastewater, and agriculture. While industry, urban development, and agriculture can cause significant changes in the landscape which can result in the degradation of fish habitat, the potential for land use change to degrade fish habitat is discussed above in the “Habitat loss” section. In this section, the focus will be on the exogenous inputs from industrial, urban, and agricultural areas that can impact fish populations.

#### Industrial

There are a variety of inputs from industry (e.g., pulp and paper, mining, metal refining, oil and gas extraction and refining) into freshwater systems that can have an adverse effect on fish populations (Guiney et al. 1987a; Muscatello et al. 2006; Hewitt et al. 2008; Marshall 2013; Cozzarelli et al. 2017). This is not meant to be an exhaustive discussion of the impact that industrial inputs can have on freshwater fish populations in North America. This section will simply highlight several of the most important.

Pulp and paper production in certain regions of North America have damaged fish populations as result of the release of untreated or insufficiently treated effluent (Munkittrick et al. 1991, 2002; Hodson et al. 1992; Basu et al. 2009). Before the 1980s, pulp and paper mills were releasing effluent with a relatively high biological oxygen demand and relatively high amounts of fiber (McMaster et al. 2006). Before regulation, these effluents were found to cause a reduction in dissolved oxygen of receiving streams and damage spawning beds for a number of fish species (McLeay and Associates 1987). In the 1980s and 1990s, regulatory changes were made to reduce the biological oxygen demand and fiber content of effluent, but effluent discharge continues to cause a reduction in egg production, delay maturity, and cause changes in second sexual characteristics of fish downstream of the mill (Munkittrick et al. 1997; McMaster et al. 2006). It was not until further effluent treatment upgrades were mandated my regulators that the impact of pulp and paper mill effluent in North America’s streams was brought under control (Kovacs et al. 2002; McMaster et al. 2006).

Along with pulp and paper effluent, inputs from a variety of types of mining (e.g., coal, gold, lead–zinc, uranium) have had significant adverse effects on fish populations in different regions across North America (Hoehn and Sizemore 1977; Muscatello et al. 2006; Schorr and Backer 2006; Allert et al. 2009; Daniel et al. 2015; Chételat et al. 2019; Martin et al. 2021). Mountaintop removal mining to extract coal in the Appalachian Mountains of the eastern USA is an example of mining that can have detrimental effects on fish populations (Hitt and Chambers 2014; Miller and Zégre 2014; Simonin et al. 2021). The process of mountaintop mining involves the removal of overlying soil and rock to expose coal seams within the mountain, while the removed overburden is disposed of in adjacent valleys (USEPA 2011a). A relatively large number of studies have consistently observed that the activities from mountaintop mining cause negative effects on fish assemblages within the catchment due to degraded water quality (Hopkins Ii and Roush 2013; Hitt and Chambers 2014; Giam et al. 2018; Martin et al. 2021; Simonin et al. 2021). A broader investigation conducted by Daniel et al. (2015) characterized the association between mining and fish assemblages in streams across three large ecoregions of the eastern USA. Increased amount of mining in a catchment corresponded with decreases in a number of fish community metrics (Daniel et al. 2015). In their study, they also found that compared to other land uses over large areas (i.e., urban land use, agriculture), mining in the catchment had a greater impact on fish assemblages (Daniel et al. 2015).

Other activities related to resource extraction that have adversely affected fish communities are oil and gas extraction, refining, and transport (Guiney et al. 1987a; deBruyn et al. 2007; Gillen and Kiviat 2012; Kimmel and Argent 2012; Marshall 2013; Cozzarelli et al. 2017). The oil and gas industry have had a greater impact on marine and coastal fish populations due to oceanic oil spills compared to freshwater fish populations (e.g., Exxon Valdez, Deepwater Horizon). Recently, the adverse effects of the oil and gas industry on freshwater fish populations in North America have been due to failures in infrastructure (e.g., pipelines rupture, train derailment, chemical storage leak). Within the oil and gas industry in North America, pipeline leaks appear to be the most common cause of adverse effects to freshwater fish populations. There are several examples across North America of pipelines releasing relatively large volumes of petroleum products or wastewater from the extraction of petroleum products into fish-bearing streams. For example, approximately 11.4 million liters of wastewater from oil production leaked from a pipeline into Blacktail Creek in North Dakota (Cozzarelli et al. 2017). Water quality downstream of the spill declined to the point where it caused endocrine disruption in fish and decreased survival (Cozzarelli et al. 2017). Another example is the release of 1310 barrels of aviation kerosene from a pipeline into the Roaring Run Creek in Pennsylvania, which resulted a significant decline in the diversity and abundance of fish downstream of the leak site (Guiney et al. 1987a, 1987b). According to the Pipeline and Hazardous Materials Safety Administration of the US Department of Transportation, the frequency of significant pipeline incidents in the USA has remained relatively stable over the last 20 years (U.S. Department of Transportation 2021). The number of pipeline incidents per year from 2001 to 2020 ranged from 341 to 719, with the total of public and industry cost per year ranging from US $166 million to 1.8 billion (U.S. Department of Transportation 2021). This would suggest that the risk of pipeline leakage to freshwater fish populations in North America is not decreasing.

As mentioned, there are a variety of other industries (e.g., manufacturing, textile) that have been responsible for the release of materials that would be deleterious to freshwater fish in North America (Degani 1945; Black et al. 1954; McKim et al. 1976; Nriagu et al. 1983). However, many of these inputs have declined with the implementation of regulations on industrial wastewater than can be released to surface waters in North America.

#### Urban

The expansion of urban land use in the North America has resulted in a significant increase in impervious surfaces across the landscape (Jennings and Taylor 2002; Nowak and Greenfield 2020). Urban land use in the USA has tripled since 1949, with impervious surfaces on the landscape following a similar trajectory (Bigelow and Borchers 2017). An increase in impervious surfaces in urban areas can have detrimental effects on surrounding streams through changes in the quality and quantity of stormwater runoff (Finkenbine et al. 2000; Morse et al. 2003; Walsh et al. 2016; Barnum et al. 2017). These changes in water quality and quantity caused by urbanization can have a detrimental effect on fish diversity and abundance in streams (Weaver and Garman 1994; Finkenbine et al. 2000; Seilheimer et al. 2007).

Additionally, urban areas produce relatively large volumes of municipal wastewater, which in most cases is deposited into aquatic ecosystems, many of those being freshwater lakes and rivers. Environment and Climate Change Canada have outlined that millions of cubic meters of wastewater are discharged from municipal systems and municipal wastewater is one of the largest sources of pollution to surface water in Canada (ECCC 2020). Before the establishment of wastewater treatment standards in North America, the biological oxygen demand of wastewater was the primary concern for freshwater ecosystems. There are a number of studies from the 1950 to the early 1980s describing the impact of untreated sewage on lakes and rivers in North America (Cross 1950; Surber 1953; Fremling 1964; Mills et al. 1966; Dominy 1973). The depletion of dissolved oxygen in freshwater systems due to the release of untreated wastewater often resulted in the mass mortality of large numbers of fish (Cross 1950; Terral 1964; Porges 1967; Staub et al. 1970; Casterlin and Reynolds 1977). As a consequence, most jurisdictions enacted legislation to mandate treatment of municipal wastewater to reduce the impact on aquatic ecosystems into which it was released (e.g., Clean Water Act in the USA) (Government of the United States 1972; Government of Canada 2012; USEPA 2022a). While the initial regulations on municipal wastewater did reduce the biological oxygen demand of effluent being released into aquatic ecosystems, effluent continued to have a more subtle impact on fish populations. A number of studies have found that even wastewater that has undergone primary and secondary treatment can cause endocrine disruption in fish, which results in a decline in exposed fish populations (Tilton et al. 2002; Vajda et al. 2008; Tetreault et al. 2011, 2013; Bahamonde et al. 2015). For example, Tetreault et al. (2013) observed lower abundance, diversity, species, and family richness in the fish communities downstream of municipal wastewater effluent discharges along the Grand River in the province of Ontario compared to fish communities upstream of the discharges. Below these same discharges, other studies have observed increased incidence of intersex and reproductive impairment in several fish species (Tetreault et al. 2011, 2021; Tanna et al. 2013; Bahamonde et al. 2015). The ability of partially treated municipal wastewater to disrupt the endocrine system of fish is not isolated the Grand River, and it has been observed across North America (Woodling et al. 2006; Vajda et al. 2008, 2011; Writer et al. 2010; Barber et al. 2011, 2015). However, other studies are showing that tertiary and other advanced forms of municipal wastewater treatment can remove many compounds contributing to endocrine disruption in fish (Barber et al. 2012; Baynes et al. 2012; Hicks et al. 2017). The challenge now is to implement these higher levels of treatment at municipal wastewater treatment facilities across North America.

#### Agricultural

The conversion of land to agriculture in the USA peaked in the 1980s and agricultural land has been in decline since peaking (Waisanen and Bliss 2002; USDA 2019). Between 2001 and 2016, 11 million acres of agricultural land in the USA has been converted to urban or residential land use (Freegood et al. 2020). Approximately 52% of the 2.26 million acres of land in the USA is used for agriculture (USDA 2019). In Canada, the percentage of land use for agriculture is considerably lower at 7.3% of the 2.28 million acres because of soil quality and climatic limitations (Statistics Canada 2014). The conversion of the natural landscape to agriculture has had an impact on the freshwater ecosystems in North America. In the “Habitat loss” section, the adverse effects of changes in land use on fish habitat (e.g., riparian structure, hydrology, sedimentation) were outlined. In this section, the focus will be on the exogenous inputs from agriculture that can have an adverse effect on freshwater fish populations, i.e., nutrients and pesticides.

##### Nutrients

Along with municipal wastewater and runoff, agricultural runoff is a major source of nutrients to surface water (Bachmann 1980; Carpenter 2008). The addition of nutrients, particularly phosphorus, to freshwater ecosystems represents a risk to fish populations as they trigger eutrophication and/or harmful algal blooms (Beeton 1965; Leach et al. 1977; Lee et al. 1978; Edmondson and Lehman 1981; Powers et al. 2005; Schindler et al. 2016). Eutrophication occurs when nutrient addition stimulates excessive algal and macrophyte growth, which leads to a reduction in dissolved oxygen when the algae and macrophyte community decay (Schindler et al. 2008; Scavia et al. 2014; Watson et al. 2016). Excess nutrients can also catalyze the growth of algal species (e.g., *Microcystis* spp.) that produce chemicals (e.g., microcystins) that are relatively toxic to fish (Edmondson and Lehman 1981; Murphy et al. 2003; Steffen et al. 2014; Watson et al. 2016). The reduction of dissolved oxygen and/or exposure to chemicals produced by harmful algal blooms can have an adverse effect on fish populations, along with other aquatic biota (Barica 1978; Cahoon et al. 1990; Smith et al. 1999; Blann et al. 2009).

The US Geological Survey’s National Water-Quality Assessment (NAWQA) found that the concentrations of total nitrogen and phosphorus were 2 to 10 times greater in US streams than recommended by the USEPA to protect aquatic life (Dubrovsky et al. 2010). The concentration of nitrate, ammonia, total nitrogen, and orthophosphate, and total phosphorus exceeded background levels in > 90% of the 190 streams draining agricultural and urban watershed that were surveyed as part of NAWQA (Dubrovsky et al. 2010). Nutrient pollution of rivers and lakes is also an issue in Canada, particularly in certain regions, e.g., southwestern Ontario. For example, the Ontario Ministry of the Environment observed that the mean concentration of total phosphorus at all the stream sites (*n* = 15) that were monitored from 2004 to 2009 in southwestern Ontario exceeded the provincial water quality objective for the prevention of eutrophication (Ontario Ministry of the Environment 2012). Rising nutrient inputs to the Laurentian Great Lakes have also been observed over the last 50 years (Burniston et al. 2018; Mezzacapo 2018; Knight and Bottorff 2020). The relationship between freshwater communities and nutrients in landscapes dominated by agriculture is complex. For example, in Indiana and Ohio, habitat variables had a greater influence on algal-diatom and fish communities compared to nutrient concentrations (Caskey and Frey 2009).

Several studies have documented the effect of eutrophication due to nutrient addition on freshwater fish communities in North America. Leach and Nepszy (1976) discuss the effect that commercial fishing and eutrophication have had on the fish community in Lake Erie. Nutrient loading played a role in the significant decline of lake trout, lake whitefish, lake herring, sauger (*Sander canadensis*), blue pike, and walleye (*Sander vitreus*) populations in Lake Erie over the last 200 years (Leach and Nepszy 1976). Eutrophication of the hypolimnion in Lake Erie during the summer caused many of these species to concentrate in the deeper water of the eastern basin of Lake Erie, which made them more vulnerable to the commercial fishery (Regier and Hartman 1973; Leach and Nepszy 1976). Eutrophication also played a role in the decline of large piscivorous fish in eastern Lake Ontario and the Bay of Quinte, which was followed by an increase in alewife and white perch (*Morone americana*) (Hurley and Christie 1977). Eutrophication can also cause acute mass mortality of fish in water bodies due to a relatively rapid drop in dissolved oxygen. For example, Hoyer et al. (2009) investigated the 407 fish kills that were reported to the Florida Fish and Wildlife Conservation Commission from 1984 to 2002. Approximately 64% of the fish kills were due to a decline in dissolved oxygen within eutrophic canals, creeks/rivers, or ponds/lakes (Hoyer et al. 2009).

Fish kills caused by harmful algal blooms, which are a result of excess nutrient additions, have also been documented across North America. Harmful algal blooms in the Upper Klamath in the US state of Oregon due to nutrient inputs is hindering juvenile recruitment of the endangered Lost River sucker (*Deltistes luxatus*) and shortnose sucker (*Chasmistes brevirostris*), which is stalling the recovery effort of these species (Burdick et al. 2020). The state of Texas has been dealing with extensive fish kills in river systems on a relatively frequent basis from harmful algal blooms (*Prymnesium parvum*) since 2001 (Sager et al. 2008; Southard et al. 2010; Brooks et al. 2011). The issue with *P. parvum* blooms was first identified when approximately 110,000 and 500,000 fish were killed from October to November in 1985 and 1986, respectively, in river systems throughout Texas by the *P. parvum* blooms (Southard et al. 2010). From 1981 to 2008, the Texas Parks and Wildlife Department was reported 153 fish mass mortality events due to *P. parvum* blooms across the river systems in the state, which corresponds to the death of 34,463,463 fish (Southard et al. 2010). The blooms threaten sixteen species of fish in Texas that are already listed as threatened with extinction (e.g., Rio Grande silvery minnow *Hybognathus amarus*, Leon Springs pupfish *Cyprinodon bovinus*, Big Bend gambusia *Gambusia gaigei*, blue sucker *Cycleptus elongatus*, Pecos pupfish *Cyprinodon pecosensis*, and Rio Grande darter *Etheostoma graham*i) (Southard et al. 2010). These blooms have also become an issue in the states of New Mexico and Oklahoma and in the Ohio river drainage (Hambright et al. 2014; Israël et al. 2014; Hartman et al. 2021).

The eutrophication of freshwater lotic and lentic ecosystems in North America, due to nutrient pollution, is having an adverse effect on the fish communities in these systems. Agriculture certainly holds a portion of the responsibility for the nutrient pollution that has been observed across the continent, especially when we consider that the dominant land use in many catchments is agriculture. However, agriculture has been able to increase their productivity without significant increases in fertilizer consumption. The productivity of agriculture in the USA has increased by 175% from 1948 to 2019 (Wang et al. 2022), while the average annual growth rate of fertilizer consumption ranges from − 3.46 to 8.52 across 48 states in the USA from 1960 to 2004, excluding Hawaii and the District of Columbia (USDA 2022). A number of states have seen a decline in fertilizer consumption (e.g., Rhode Island, Massachusetts), while others have seen a considerable increase (e.g., South Dakota, Indiana, Kansas) (USDA 2022). Not all of the responsibility should not be laid at the feet of agriculture, urban land use can also be a significant contributor to nutrient pollution in freshwater ecosystems (Frei et al. 2021). As mentioned earlier, agricultural land use in the USA has been steady decreasing since the 1980s and mainly being replaced by urban and residential land use (Freegood et al. 2020). The USGS reported that the greatest measured concentrations of total phosphorus and total nitrogen were observed in small streams not only draining agricultural lands but also urban land (USGS 1999). Phosphate derived from household detergents released from municipal wastewater treatment facilities was a major driver of lake eutrophication, which resulted in a ban on phosphates in household detergents (Hartig and Horvath 1982; Schindler et al. 2016). However, municipal wastewater treatment facilities continue to be a major source of nutrients to freshwater ecosystems (USGS 1999; Carey and Migliaccio 2009).

##### Pesticides

Along with nutrients, pesticides are a potential pollutant to freshwater ecosystems from agriculture. However, it is important to acknowledge that there are other potential sources of pesticides to freshwater, e.g., forestry, vegetation management, landscaping/lawn care, aquaculture, management of invasive species, and protection of public health (Ramwell et al. 2004; Overmyer et al. 2005; Thompson 2011; Van Geest et al. 2014; Schoch-Spana et al. 2020; Robichaud and Rooney 2021). Consequently, in this section, the focus will be on the potential effect of pesticides on freshwater fish populations across various uses.

Pesticides are designed to elicit adverse effects on biota that have been identified as pests. Consequently, there is the potential for this group of chemicals to have an adverse effect on non-target biota, including fish. However, over the last 80 years, the toxicity and bioaccumulative potential of new classes of pesticides in fish has markedly declined. The rigor of the regulatory system for pesticides in North America has also significantly changed over the last 80 years (Graham 2019; CropLife Canada 2022; USEPA 2022b). While pesticides can pose a hazard to fish, these changes in chemistry and regulation have greatly reduced the potential risk pesticides pose to fish in North America. The groups of pesticides that pose the greatest potential risk to fish are insecticides (Munn and Gilliom 2001) due to greater conservation of target biochemical pathways between fish and insects compared to the target biochemical pathways for herbicides and fungicides (Fulton et al. 2013). For this reason, the discussion of pesticide risk to fish diversity will be focused on insecticides.

##### Relative toxicity of insecticide classes

There are 2000-year-old records describing the use of various substances to protect crops and homes from pests. In the fourth century BCE, Democritus describes the soaking of seeds in the juice of houseleek (*Sempervivum* spp.) to increase yield (Smith and Secoy 1975). Both Varro in the first century BCE and Columella in the first century CE described the use of an extract from wild cucumber (*Cucumis hystrix*) as a treatment for bed bugs (Smith and Secoy 1975). Since classical antiquity, humanity has continued to discover inorganic and organic substances that can be used to control pests in the field and in the home with varying degrees of efficacy (Unsworth et al. 2019).

An increasing body of knowledge on organic chemistry resulted in major advancements in the development of pesticides starting in the middle of the twentieth century (Müller 2002). However, these early classes of organic pesticides have relatively high toxicity towards fish, particularly insecticides (Fulton et al. 2013). Spills or improper application of certain early organic insecticides (e.g., organochlorine insecticides) has been responsible for events of mass fish mortality across North America. Between 1970 to 1977, the US Environmental Protection Agency recorded 60 and 74 fish kill events due to the organochlorine insecticides endrin and toxaphene, respectively (USEPA 1980e). Pesticides were responsible for the loss of 6–14 million fish per year in the USA from 1977 to 1987 (Pimentel et al. 1992). An important evolution in insecticide development over the last 30 years has been the design of chemicals with greater specificity for controlling insect pests and lower toxicity to non-target biota, e.g., fish (Katz 1961; Pickering et al. 1962; Anderson et al. 2015; Marlatt et al. 2019). The persistence of insecticides in freshwater ecosystems and the recommended rate of application in agriculture have also declined over the last 80 years (Eichelberger and Lichtenberg 1971; Anderson et al. 2015). A significant reduction in the number of events in which pesticides have caused mass mortality of fish has also occurred over the last 20 years (ProPharma 2019). Consequently, the risk of insecticides to freshwater fish populations has declined with the progressive development of new classes (e.g., neonicotinoid, diamide, butenolide) (Anderson et al. 2015; Finnegan et al. 2017; Jia et al. 2020).

As a case study, the acute and chronic toxicity of insecticide classes to the standard fish model species (e.g., rainbow trout *Oncorhynchus mykiss*, fathead minnow *Pimephales promelas*, etc.) have been compiled in Table 1. The newer classes of insecticides, neonicotinoids, are at least three orders of magnitude less toxic to fish among the classes of insecticides used over the last 80 years (Table 1). Generally, acute and chronic toxicity to fish have improved with each successive generation, with the exception of the pyrethroids, which demonstrate the greatest chronic toxicity among all classes of insecticides. Accumulation of pesticides in the tissue of fish (bioaccumulation) and the increase in tissue concentrations with an increase in trophic level (biomagnification) have been an issue in the past with organochlorine pesticides (Evans et al. 1991; Suedel et al. 1994; Johnston et al. 2002). Based on the octanol–water partition coefficient (Kow), it is unlikely that neonicotinoids will accumulate in the tissues of fish, which would also eliminate the possibility of biomagnification of this class within an aquatic ecosystem (Table 1). Although soil persistence has significantly decreased through successive generations of insecticides (see Brain and Anderson 2020), it must be acknowledged that aquatic persistence has remained relatively consistent, which is a consideration that pesticide manufacturers/developers should seek to address in the future. The physicochemical properties of several organochlorine pesticides also make them amenable to long-range transport, resulting in bioaccumulation and biomagnification in distant ecosystems where the pesticides are not used, e.g., arctic) (Muir et al. 1990; Kidd et al. 1993, 1995). Long-range transport and accumulation in distance aquatic ecosystems does not occur with the classes of insecticides developed in the later part of the twentieth century (Table 1).

**Table 1:** Fish and aquatic invertebrate toxicity (acute and chronic), chemical property data and application rates for classes of pesticides in chronological order of introduction

Class/compound	Fish acute LC_50_ (µg/L)	Fish chronic NOEC (µg/L)*	Aquatic invertebrate acute LC_50_ (µg/L)	Aquatic invertebrate chronic/sub-chronic NOEC (µg/L)	Log K_ow_	Aerobic aquatic half-life (d)	Anaerobic aquatic half-life (d)	Max single application rate for agriculture (lb a.i./A)	Source(s)
***Organochlorines* (*1940s to 1972*)**									
DDT	0.6	0.74	0.18	NA	7.48	28 to 56	NA	1.2 to 12	(USEPA 1980b; d; WHO 1989; Blaylock 2005)
Aldrin	2.2	NA	8	NA	6.50	~ 28	NA	0.5 to 5	(USEPA 1980d; a; 1986; 2003)
Dieldrin	2.5	0.22	4.5	0.73	6.20	> 112	NA	0.1 to 1.5	(Sharom et al. 1980; USEPA 1980a, 2003; ATSDR 2002)
Heptachlor	7.4	0.86	0.9	12.5	5.44	3.5 to 400^b^	NA	2.5 to 10	(USEPA 1960; Macek et al. 1976; USFWS 1980; ATSDR 1989; 2007)
Toxaphene	2.0	< 0.039	10	0.07	6.64	< 42	< 42	1.5 to 2	(USEPA 1975; 1980c; USFWS 1980; Lacayo et al. 2004; ATSDR 2014)
Mirex	> 100	2	> 1	< 2.4	5.28	> 56	> 56	0.001 to 0.004	(USFWS 1980; Buckler et al. 1981; Sanders et al. 1981; Skaar et al. 1981; ATSDR 2019)
Chlordane	36.9	< 0.32	28.4	0.7	5.54	7	NA	1.5	(USEPA 1969; 1977; Oloffs et al. 1978)
Lindane	1.7	2.9	10	54	3.72	3 to 300	< 112	0.4 to 3	(USEPA 2002; ATSDR 2005)
***Organosphosphates* (*1960s to present*)**									
Chlorpyrifos	1.8	0.57	0.0138	0.005	4.70	30.5	50.2 to 125	0.3 to 6	(USEPA 2006c; 2018a)
Malathion	33	21	1.0	0.06	2.80	0.5 to 10	2.5	0.156 to 7.5	(USEPA 2009c; 2018c)
Diazinon	90	< 0.55	0.20	0.17	3.77	6.3 to 41.0	24.5	0.25 to 5	(USEPA 2006d; 2018b)
Methyl parathion	1850	< 80	0.97	0.25	2.86	12.3	33.3	0.5 to 2	(USEPA 2008; 2009b)
Dichlorvos	183	5.2	0.07	0.0058	1.58	NA	NA	0.2	(USEPA 2006a)
Phosmet	70	3.2	2	0.8	2.95	NA	NA	0.7 to 6	(USEPA 2006e; 2009a)
Azinphos-methyl	1.2	0.44	0.16	0.25	2.75	NA	NA	0.125 to 2.5	(USEPA 2005; 2006b)
Fenitrothion	1720	46	2.3	0.087	3.319	NA	0.82	0.3125 to 3	(USEPA 1995; FAO 2003)
***Pyrethroids* (*1970s to present*)**									
Bifenthrin	0.15	0.1	0.000493	0.00005	6.40	92.9 to 276	587 to 618	0.04 to 0.5	(USEPA 2010a; 2016b)
Permethrin	0.79	NA	0.0066	0.0042	6.10	5.37 to 10.5	86	0.007 to 0.4	(USEPA 2011b; 2016b)
Lambda-Cyhalothrin	0.078	0.031	0.0003	0.00022	7.00	21.1 to 52.9	57.7 to 6320	0.03 to 0.156	(USEPA 2010b; 2016b)
Cypermethrin	0.39	0.051	0.00056	< 0.00005	6.40	9.5	17.7	0.025 to 0.1	(USEPA 2012a; 2016b)
Deltamethrin	0.15	0.017	0.0002	0.000026	5.4	9.29 to 120	60.7 to 98.9	0.009 to 0.033	(USEPA 2016b)
Fenpropathrin	2.2	0.06	0.00305	< 0.0015	5.1	88.7 to 618	67 to 1250	0.1 to 0.4	(USEPA 2010c; 2016b)
Cyfluthrin	0.209	0.010	0.025	0.00012	6	8.44 to 44.8	9.41 to 26.2	0.0044 to 0.07	(USEPA 2010b; 2016b)
Esfenvalerate	0.142	0.017	0.000848	0.0007	75	17.2 to 48.2	11.5 to 73.7	0.05 to 0.19	(USEPA 2016b)
***Neonicotinoids* (*1990s to present*)**									
Thiamethoxam	> 111,000	1700	35	0.74	− 0.13	16.3 to 35.1	20.7 to 28.6	0.17 to 0.27	(USEPA 2017e)
Imidacloprid	> 100,000	9,000	0.77	0.01	0.57	236	33	0.043 to 0.25	(USEPA 2017b)
Clothianidin	> 91,400	9,700	22	< 0.05	1.12	187	81	0.05 to 0.2	(USEPA 2017a)
Dinotefuran	> 99,100	6,360	790	< 0.044	− 0.549	58.9 to 61.2	77.2	0.068 to 0.54	(USEPA 2017c)
Acetamiprid	100,000	19,200	66	2.5	0.8	86.6 to 96.2	477 to 585	0.03 to 0.52	(USEPA 2017d)
Sulfoxaflor	266,000	650	640	19	< 1	37 to 88	103 to 382	0.012 to 0.133	(USEPA 2019)
Thiacloprid	19,700	918	31.3	1.1	1.26	11 to 27	183 to 939	0.088 to 0.25	(USEPA 2012b)

*NA*, no risk assessment value available in the literature. *Where freshwater species data was lacking estuarine/marine species (e.g., the sheepshead minnow) was used. ^b^Heptachlor epoxide

##### Incident reporting data

**Fig. 3:**
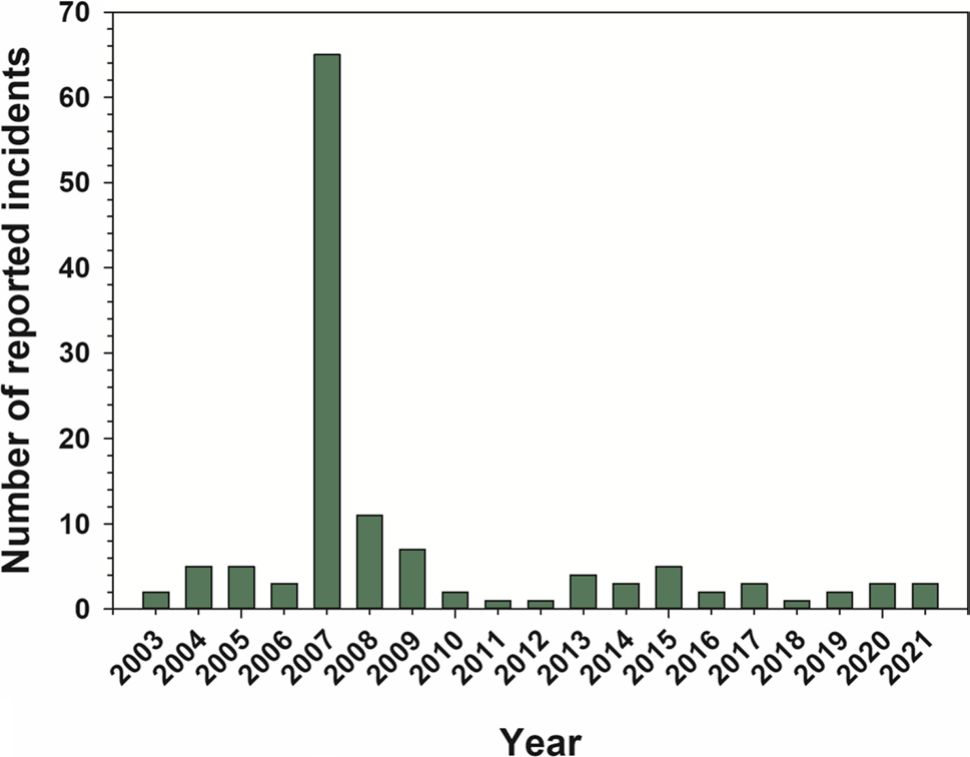
The number of incidents of fish mortality due to pesticide application reported in the USA each year between January 2003 and December 2021 to the ProPharma Group

**Fig. 4:**
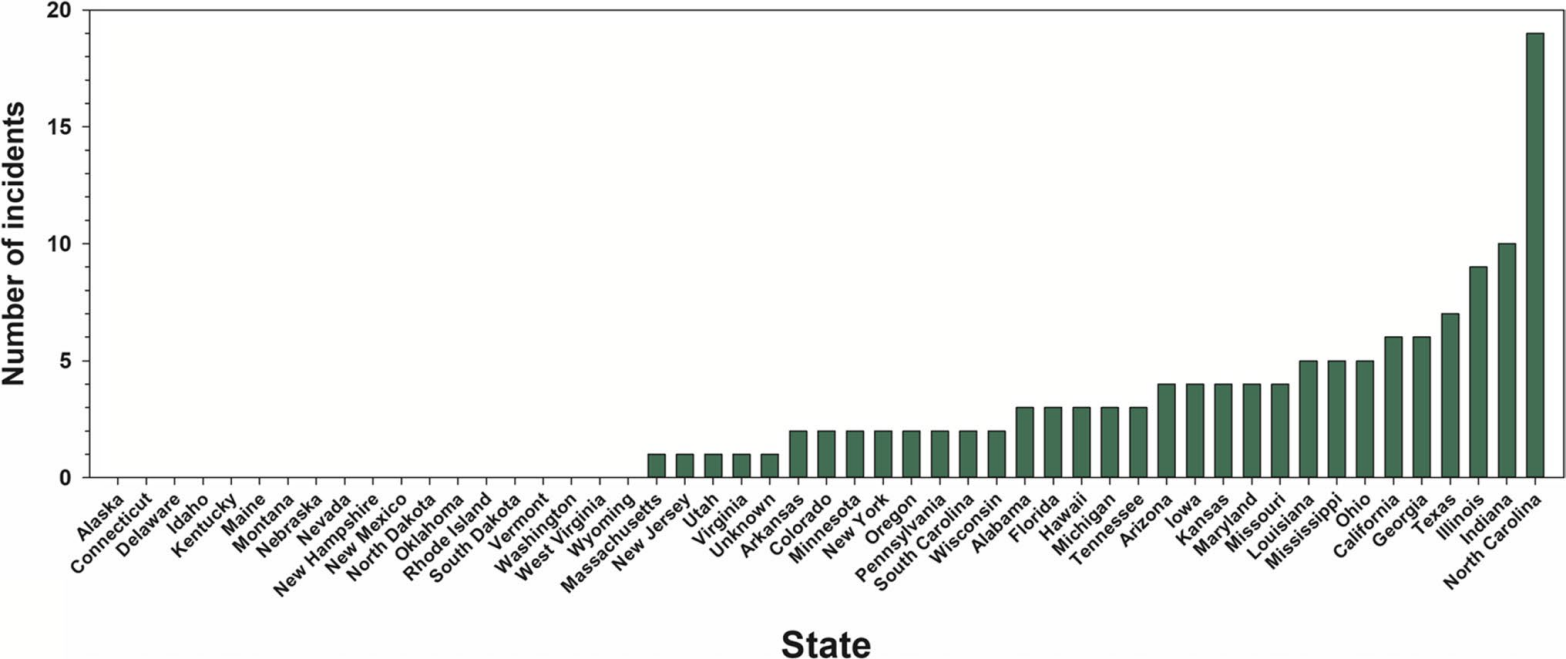
The number of incidents of fish mortality due to pesticide application by state in the USA between January 2003 and December 2021 reported to ProPharma Group

As mentioned above, spills or applications that did not follow best management practices of the classes of pesticides developed in the mid-twentieth century were found to cause mass mortality of fish (USEPA 1980a, b, c, d, e; Pimentel et al. 1992). The most recent decades have seen a considerable decline in the number fish kills due to pesticides. Mortality incident trends in the USA were explored by mining the ProPharma Group database (ProPharma 2019) between January 2003 and December 2021 for reported incidents involving freshwater fish. Collectively, 128 incidents were reported during this interval; however, the reporting was not consistent across years (Fig. 3). Of the 19 years included in the database, less than five incidents were reported per year for most years except for 2007, 2008, and 2009. An anomalously high number of incidents was reported in 2007 (65), representing half of all entries, with 11 and 7 recorded in 2008 and 2009, respectively. The surge in incident reporting in 2007 is puzzling, though, a similar pattern has been observed previously for mammals (Brain and Anderson 2020). Moreover, among the incidents reported in 2007 were two significant events in North Carolina affecting an estimated 300,000 and 100,000 fish, respectively, both attributed to pyrethroid insecticides. However, many reported incidents do not specify the number of individuals affected, and those that do are typically estimates. Consequently, it is not possible to provide a cumulative number of fish mortalities ascribed to pesticide exposures over time. The peculiar number of incidents reported in 2007 could have been the result of special interest group campaigns, exceptional circumstances (e.g., cropping and weather conditions) or other factors, but the source is ultimately speculative. Under-reporting in all other years (2003 to 2021) is also a possibility, though the singular nature of the irregularity, 1 in ~ 20 years, suggests under-reporting would have to be systematic in nature (except for a single year). Notwithstanding 2007, annual fish mortality incidents suspected of being caused by pesticide exposure are consistent. Insecticides were cited in ~ 50% of incidents, herbicides in ~ 30%, fungicides in ~ 10%, and the other ~ 10% were unknown. Most often, the route of exposure was unknown (i.e., not witnessed), so care must be taken in interpreting the incident data because exposure to the compound(s) of interest cannot always be confirmed, rather suspected based on coincidental timing, anecdotal observations, etc. Examination of the incident data by state did not reveal any obvious correlated trends in terms of land use, population, geography, or other factors (Fig. 4). The greatest number of reported incidents was attributed to North Carolina (19); nineteen states reported no incidents and 45 states reported less than 3 incidents.

##### Indirect effect on fish

The newer classes of pesticides, based on their toxicity to fish and probability of exposure, are not likely to have a direct effect on fish populations (Gibbons et al. 2015; Finnegan et al. 2017). However, there is concern that these newer classes of pesticides could have a significant effect on other species (e.g., primary producers, zooplankton, insect larvae) within a freshwater ecosystem on which fish populations rely (Fleeger et al. 2003). For example, a neonicotinoid insecticide may not have a direct effect on the fish populations within a river or lake, but there may be a direct effect on the aquatic invertebrate community which is an important source of food for certain fish species (Boyle et al. 1996). As would be expected, all major classes of insecticides used since the 1940s have considerably greater toxicity towards aquatic invertebrates compared to fish (Table 1). While in theory, pesticides could have indirect effect on fish communities, the important question is whether they cause these indirect effects at concentrations that are being observed in the surface waters of North America? The answer to this question for neonicotinoids has been provided by the recent special reviews that have been conducted by the US Environmental Protection Agency (USEPA) and the Canadian Pest Management Regulatory Agency (PMRA) (USEPA 2020; PMRA 2021b; a). The PMRA conducted a special review of the risk that the neonicotinoids clothianidin and thiamethoxam pose specifically to aquatic invertebrates (PMRA 2021a; b). At the end of the intensive special review process, PMRA determined that some conditions of use needed to be amended to reduce the potential risk of chronic toxicity of both thiamethoxam and clothianidin to aquatic invertebrates (PMRA 2021a; b). The USEPA also proposed a number of actions to mitigate the potential risk of thiamethoxam and clothianidin to aquatic invertebrates, i.e., spray drift reduction, prevent runoff, vegetative filters strips, and reduce perimeter treatment applications (USEPA 2020). The answer to the proposed question is that with minor changes to use conditions and modifications to best management practices, the risk of neonicotinoids causing indirect effects on freshwater fish in North America is *de minimis*. These special reviews conducted by the PMRA and the USEPA are examples of the rigorous review process that is in place for pesticides in North America. This same rigor is leveled at other groups of pesticides that are registered for use in North America. Consequently, the risk of indirect effects on freshwater fish across the groups of pesticides is *de minimis*. As mentioned earlier, pesticides can be hazardous to aquatic biota, but the regulatory framework ensures that the risk, i.e., probability of adverse effects, remains below an acceptable level.

For aquatic biota, exposure to, and corresponding risks from, coincidentally encountering multiple pesticides (i.e., mixtures) is a concept that may warrant further investigation. Coincident exposure of aquatic ecosystems to pesticide mixtures has been observed throughout North America and these mixtures could potentially pose a risk to fish populations (Laetz et al. 2009; Nowell et al. 2018; Raby et al. 2022). However, the probability of direct toxicity is generally low due to the relatively low toxicity of contemporary pesticide classes to fish (Nowell et al. 2018; Raby et al. 2022). Moreover, incidence of synergy is rare, and toxicity is typically driven by a single pesticide active ingredient (Cedergreen 2014; Belden and Brain 2018). Potential indirect effects through impacts on aquatic invertebrates are arguably a greater potential risk to fish populations. However, the lack of data regarding the frequency that mixtures of pesticides exceed acceptable effect levels across North America presents a challenge to quantifying corresponding risks to fish communities. Monitoring of pesticide residues in areas prone to greater concomitant exposure should be a priority to effectively assess this question regarding potential risks of pesticide mixtures to freshwater ecosystems. However, when assessing such risks to fish and other freshwater biota, additivity is generally the best model to characterize the toxicity of pesticide mixtures (Belden and Brain 2018). A number of studies have discussed the potential for synergistic interactions when exposed to multiple pesticides, but this type of interaction has been shown to be rare, particularly at concentrations observed in the environment (Cedergreen 2014).

### Recovery

While various anthropogenic activities have contributed to extensive declines in freshwater fish populations throughout North America, there have been recent efforts to reverse these trends. For example, in Canada, the Fisheries Act received royal assent and become law in 1985 (DFO 2022a). The Fisheries Act has been amended several times over the last 27 years. The most recent amendment in 2019 strengthened protections for freshwater and marine fish populations, fish habitat, and biodiversity in Canada. Throughout North America, projects have been initiated to actively restore fish habitat. For example, a project to restore the river ecosystem along 17.7 km of the Los Angeles River has begun. The project intends to restore the natural ecological and physical processes within the river (City of Los Angeles 2022). Barriers to freshwater fish populations are also being removed throughout North America. Since 1912, 1797 dams have been removed in the USA. In 2020, 69 dams were removed in 23 US states, which equates to the reconnection of over 1000 km of upstream river for fish (American Rivers 2022). Effort has also been made to control the introduction of new invasive species and to the control the populations of established invasive species. Control of the sea lamprey in the upper Great Lakes through a joint program with the US Fish and Wildlife Service and the Canadian Department of Fisheries and Oceans is a success story for invasive species control. The control program has caused a 90% reduction in sea lamprey populations (DFO 2022b). Hopefully, continued conservation and habitat restoration efforts can stem the decline in fish populations and biodiversity, and lead to recovery of populations and biodiversity.

## Conclusions

Both the Living Planet Index for Migratory Freshwater Fish, the first comprehensive global report on the status of migratory fish, and the International Union for Conservation of Nature’s Freshwater Biodiversity Unit and Freshwater Fish Specialist Group concluded that the most important threats to freshwater fish communities are habitat degradation, loss, or change, invasive species, and climate change (Arthington et al. 2016; Darwall and Freyhof 2016; Deinet et al. 2020) (Table 2). Furthermore, Mandrak and Cudmore (2010) reported that central drivers of the global extinction of 3 species, local extirpation of 18 species, and endangerment of 82 fish species in the Laurentian Great Lakes is habitat alterations, aquatic invasive species, and overexploitation (Fig. 2). There is consensus among experts that the primary drivers of previous, continued, and future declines of freshwater fish in North America are habitat alteration, dams/obstructions, invasive species, overexploitation, and climate change. Four criteria that could be used to describe the scale and magnitude of a driver’s contribution to the decline of freshwater fish are geographical scale, temporal scale, significance to viability, and ease of remediation. A scoring system could be applied to each of these criteria to further define the scale and magnitude of a driver. Table 3 presents proposed relative scoring for the drivers of fish decline in North America outlined in this article. Although arguably subjective in nature, the scoring of these criteria supports the ranking consensus on major drivers of fish decline descried above and is intended to inspire discussion and debate. The criteria were intended to capture geographic and temporal scales of influence, the severity of the influence on fish species, reversibility, and association with other drivers (i.e., relationship with, dependence on, or contribution to other drivers).

**Table 2:** Drivers of decline in freshwater fish communities and freshwater ecosystems that have been identified at the regional to global scale by various groups of researchers

The Living Planet Index for Migratory Freshwater Fish (Deinet et al. 2020)	International Union of Conservation of Nature – Freshwater Biodiversity Specialist Group (Arthington et al. 2016; Darwall and Freyhof 2016)	United States Environmental Protection Agency National Rivers and Streams Assessment 2008–2009^a^ (USEPA 2016a)	Scientists’ Warning to Humanity: Rapid Degradation of the World’s Large Lakes^b^ (Jenny et al. 2020)	Rapid Decline of California’s Native Inland Fishes: A Status Assessment (Moyle et al. 2011)	The fall of Native Fishes and the rise of Non-native Fishes in the Great Lakes Basin (Mandrak and Cudmore 2010)
North America	Global	USA	Global	State of California	Laurentian Great Lakes
1.Habitat degradation, loss, or change 2.Invasive species 3.Pollution 4.Climate change 5.Exploitation	1.Invasive species 2.Climate change 3.Habitat degradation, loss, or change 4.Pollution 5.Exploitation	1.Total phosphorus 2.Total nitrogen 3.Riparian vegetation cover 4.Riparian disturbance 5.Excess sedimentation 6.In-stream fish habitat 7.Salinity 8.Acidification	•Increased nutrient loading •Climate change •Acidification •Exploitation •Habitat degradation, loss, or change •Invasive species	1.Major dams 2.Estuarine alteration 3.Agriculture 4.Urbanization 5.Transportation 6.Harvest 7.Alien species 8.Grazing	1.Habitat alterations 2.Aquatic invasive species 3.Exploitation

^a^Attributable Risk to Fish Multi-metric Index; index based on a variety of metrics, including taxonomic richness, taxonomic composition, pollution tolerance, habitat and feeding groups, spawning habits (specifically, the percent of individuals that deposit eggs on or within the substrate in shallow waters), the number and percent taxa that are native. ^b^The authors did not rank the stressors that they identified as widespread and with strong impacts. The stressors are listed in the order that they appeared in the article

**Table 3:** The potential drivers of freshwater fish decline in North America and criteria and associated scoring system to describe the scale and magnitude of driver’s contribution to decline. This table was based on information that has been previously presented in the literature (see Table 2) and the expert opinions of the authors. The purpose of the table is to be a point of departure for discussion about prioritizing of the drivers of freshwater fish decline in North America

Criteria	Geographic scale	Temporal scale	Significance to viability	Ease of remediation	Associated with other drivers	Percent score
	1 regional to 5 national	1 short term to 5 long term	1 minor to 5 catastrophic	1 easy to 5 very difficult	1 not associated to 5 highly associated	(Sum / 25) × 100
**Potential drivers**						
Habitat loss	5	5	5	4	5	96
Dams/obstructions	5	5	4	4	4	88
Invasive species	4	4	4	4	2	72
Overexploitation	3	3	3	3	2	56
Climate change	5	5	5	5	5	100
*Pollution*						
Industrial	2	3	3	3	4	60
Urban	2	3	3	3	4	60
Agricultural						
Nutrients	4	3	4	3	4	72
Pesticides	2	2	2	3	3	48

The prevention of pollution in the form of nutrients and toxic chemicals certainly needs to be considered to prevent further decline of freshwater fish populations and support their recovery. However, based on the available data regarding anthropogenic factors driving freshwater fish decline, and considering the rigorous regulatory framework in place, pesticides are not a significant driver of freshwater fish declines relative to other more prominent drivers. Consequently, we suggest that public attention should more accurately and judiciously align with the relative importance of primary drivers contributing to freshwater fish declines and conservation investment should continue to be appropriated accordingly (USFWS 2022). When put into context among the myriad of drivers contributing to freshwater fish declines, a disproportionate amount of concern has focused on pesticides. An example of this disproportionate concern is an article published in the prestigious journal Science that reported neonicotinoid insecticides caused a significant decline in zooplankton abundance, which led to the collapse of several fisheries (Yamamuro et al. 2019). Despite considerable deficiencies in methodology and interpretation of data, the study was published in Science and the study was the central focus of an article published by the magazine National Geographic with the title “How the world’s most widely used insecticide led to a fishery collapse” (Main 2019). National Geographic had a cross-platform audience of 40.9 million in 2017 (Richter 2018). Yet, a simple evaluation of the conclusion presented by Yamamuro et al. (2019) using the Bradford-Hill criteria for causation would have revealed the flaws. Moreover, in June 2022, the US EPA released the final Biological Evaluation (BE) for thiamethoxam, concluding “likely to adversely affect” effects determinations for over 90% of threatened and endangered (“listed”) fish species considered in the analysis (USEPA 2022a, b, c). However, it is consequential to point out that the BE process implemented by EPA is not only extremely conservative and precautious by design but also autonomous of all other potential drivers contributing to listed species status outcomes. Thus, pesticide BE determinations are essentially made in a vacuum, often projecting ominous conclusions with little to no context regarding the broader macrocosm of potential factors consequential to species viability.

Ultimately, to address the initial question posed in the title of this thesis (how do agricultural pesticides compare to other drivers?), among the macro drivers contributing to freshwater fish declines, agricultural pesticides rank low, yet, with respect to research focus and media coverage pesticides feature prominently. Pesticides have a legal construct that is relatively easy to exploit, encouraging litigious opportunism. Pesticides are conveniently easy to test, resulting in experimental proliferation of data, varying in quality and relevance, which unfortunately can incentivize sensationalism to capture the public’s imagination. Finally, the application of pesticides is highly visible. There is no denying the indelible legacy of the organochlorines, which persists even today, or the potential for successive classes of insecticides to impact freshwater fish; those facts are not in dispute. The point illustrated here is that, when framed in the context of other factors contributing to freshwater fish declines, agricultural pesticides are not a major driver. Moreover, contemporary classes of insecticides such as the neonicotinoids are orders of magnitude less toxic to fish than their predecessors. The agricultural landscape is shrinking while the global population is expanding (exponentially). Concurrently, calls for conservation are growing as rural populations are declining. Perhaps we should be more judicious in how we frame pesticides and agriculture in general, because without farming and the technology that underpins modern food production, growing more from less will not be possible.

## Data Availability

All data were sourced from the primary literature and cited accordingly in the manuscript.
